# Supplementary material for: Alkali metal bilayer intercalation in graphene
Source: Nat Commun. 2024 Jan 24;15:425. doi: 10.1038/s41467-023-44602-3 (PMC11258350; doi:10.1038/s41467-023-44602-3)
Supplement: Supplementary file 1 — Supplementary Information [file 41467_2023_44602_MOESM1_ESM.pdf]

Supplementary Information

Alkali Metal Bilayer Intercalation in Graphene

Yung-Chang Lin[1,2]*, Rika Matsumoto[3], Qiunan Liu[2], Pablo Solís-Fernández[4], Ming-Deng Siao[5], Po-Wen Chiu[5,6], Hiroki Ago[4,7], Kazu Suenaga[1,2]*

[1]Nanomaterials Research Institute, National Institute of Advanced Industrial Science and Technology (AIST), Tsukuba 305-8565, Japan

[2]The Institute of Scientific and Industrial Research (ISIR-SANKEN), Osaka University, Osaka 567-0047, Japan

[3] Department of Engineering, Tokyo Polytechnic University, 5-45-1 Iiyamaminami, Atsugi, Kanagawa 243-0297, Japan

[4] Global Innovation Center (GIC), Kyushu University, Fukuoka 816-8580, Japan

[5]Department of Electrical Engineering, National Tsing Hua University, Hsinchu 30013, Taiwan

[6]Institute of Atomic and Molecular Sciences, Academia Sinica, Taipei, 10617, Taiwan

[7] Interdisciplinary Graduate School of Engineering Sciences, Kyushu University, Fukuoka 816-8580, Japan

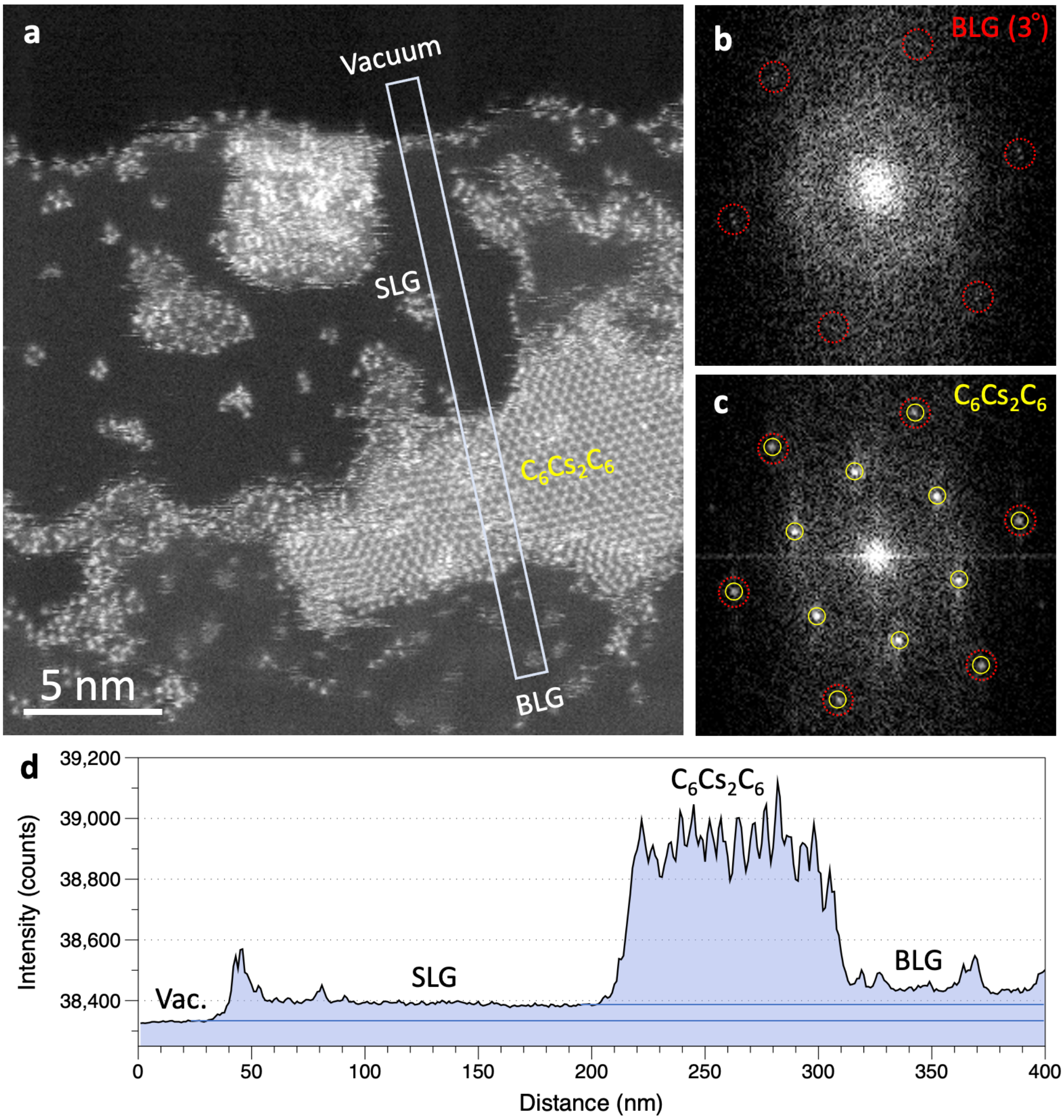

Supplementary Fig. 1 | STEM characterization of BLG and intercalated Cs layer. (a) ADF image of Cs intercalation in BLG. (b) FFT image from the BLG region in which the twist angle of BLG is 3°. (c) FFT image from the region of $C_6Cs_2C_6$. (d) ADF profile from the white box in (a). The ADF intensity of BLG is double that of SLG.

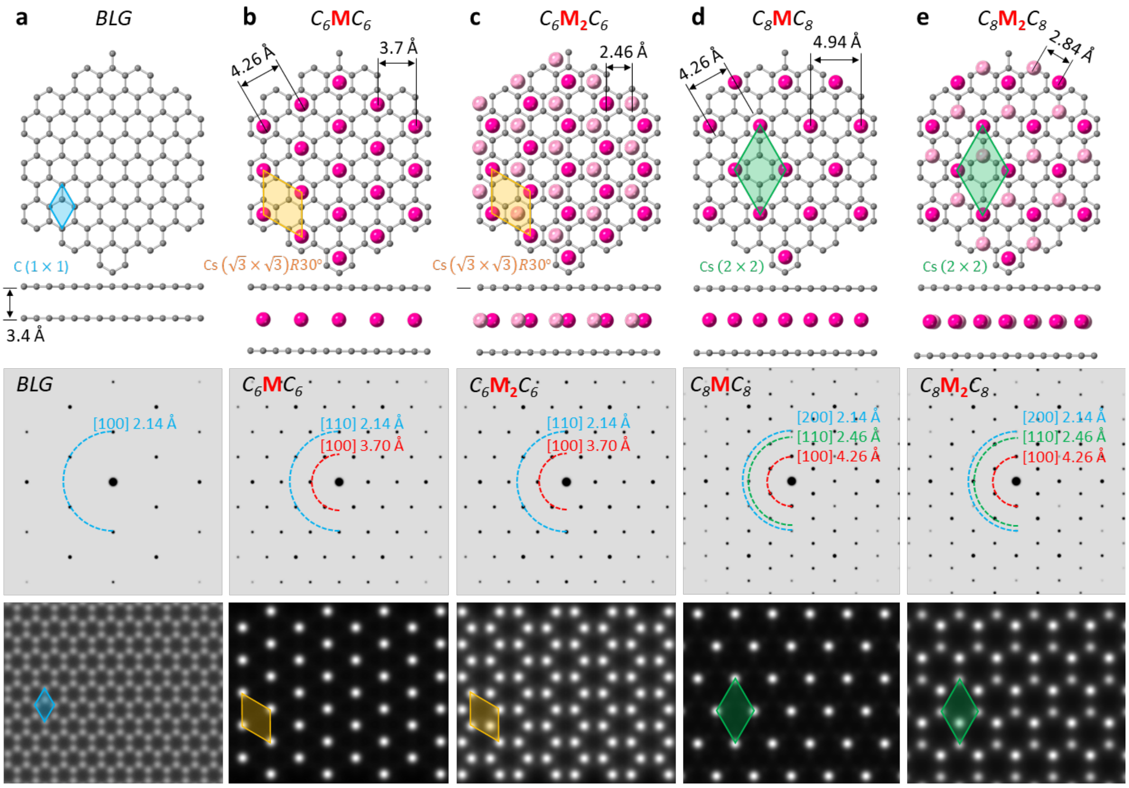

Supplementary Fig. 2 | Atomic structure, diffraction pattern, and STEM image simulation of BLG, C_6MC_6, $C_6M_2C_6$, C_8MC_8, $C_8M_2C_8$ structures. Graphene shows honeycomb shape which is hexagonal lattice with two-atom basis in single layer. The atomic model and electron diffraction pattern and STEM simulation of **(a)** bilayer graphene, **(b)** C_6CsC_6, where the orange rhombus illustrates the in-plane unit cell for the Cs $(\sqrt{3} \times \sqrt{3})R30^\circ$ structure, **(c)** $C_6Cs_2C_6$, where the Cs $(\sqrt{3} \times \sqrt{3})R30^\circ$ unitcell contains two Cs atoms, **(d)** C_8CsC_8, where the green rhombus displays the in-plane unit cell for the Cs (2×2) structure, **(e)** $C_8Cs_2C_8$, where the Cs (2×2) unit cell contains two Cs atoms. The side view model is not geometry optimized.

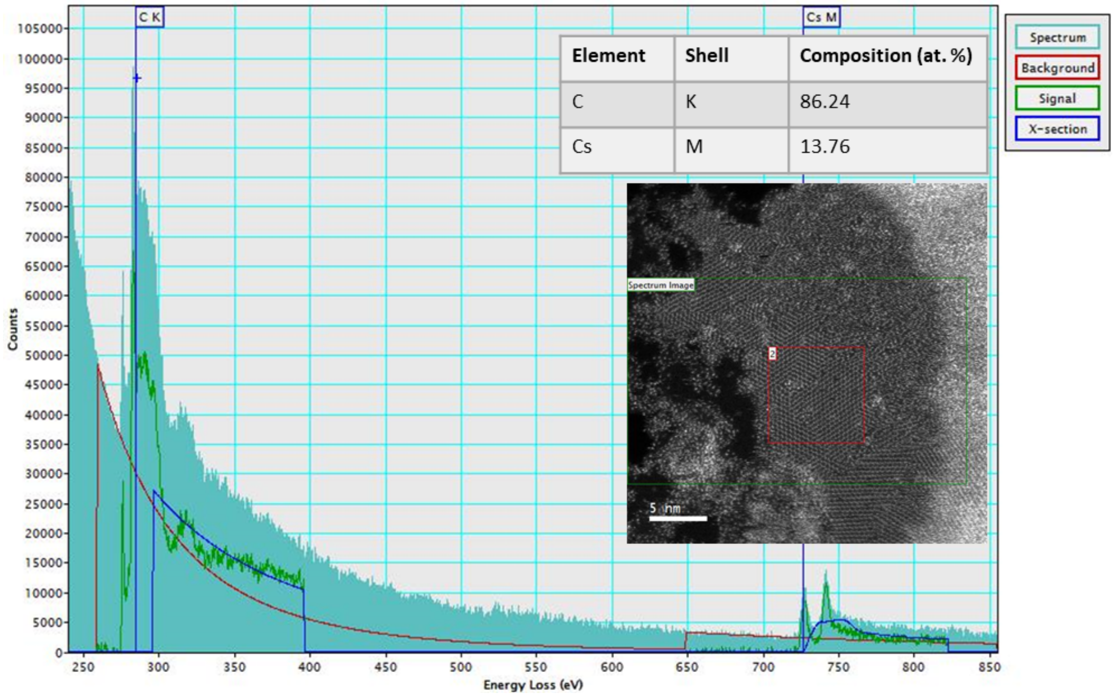

Supplementary Fig. 3 | EELS quantification for the $C_6Cs_2C_6$ structure. The EELS quantification was performed using Gatan Digital Micrograph software. The inset shows the ADF image of the Cs intercalation in BLG, with the green box representing the selected area for EELS mapping and the red box indicating the selected area for EELS quantification. Background subtraction using the Power Law model was applied to the red curve. For quantification, hydrogenic cross-section was used for the carbon K-edge, and Hartree-Slater with white line correction was used for the Cs M-edge. The selected signal window was 100 eV. The calculated atomic ratio for C is 86.24 at.%, and for Cs, it is 13.76 at.%, which is consistent with the proposed model of the $C_6Cs_2C_6$ structure.

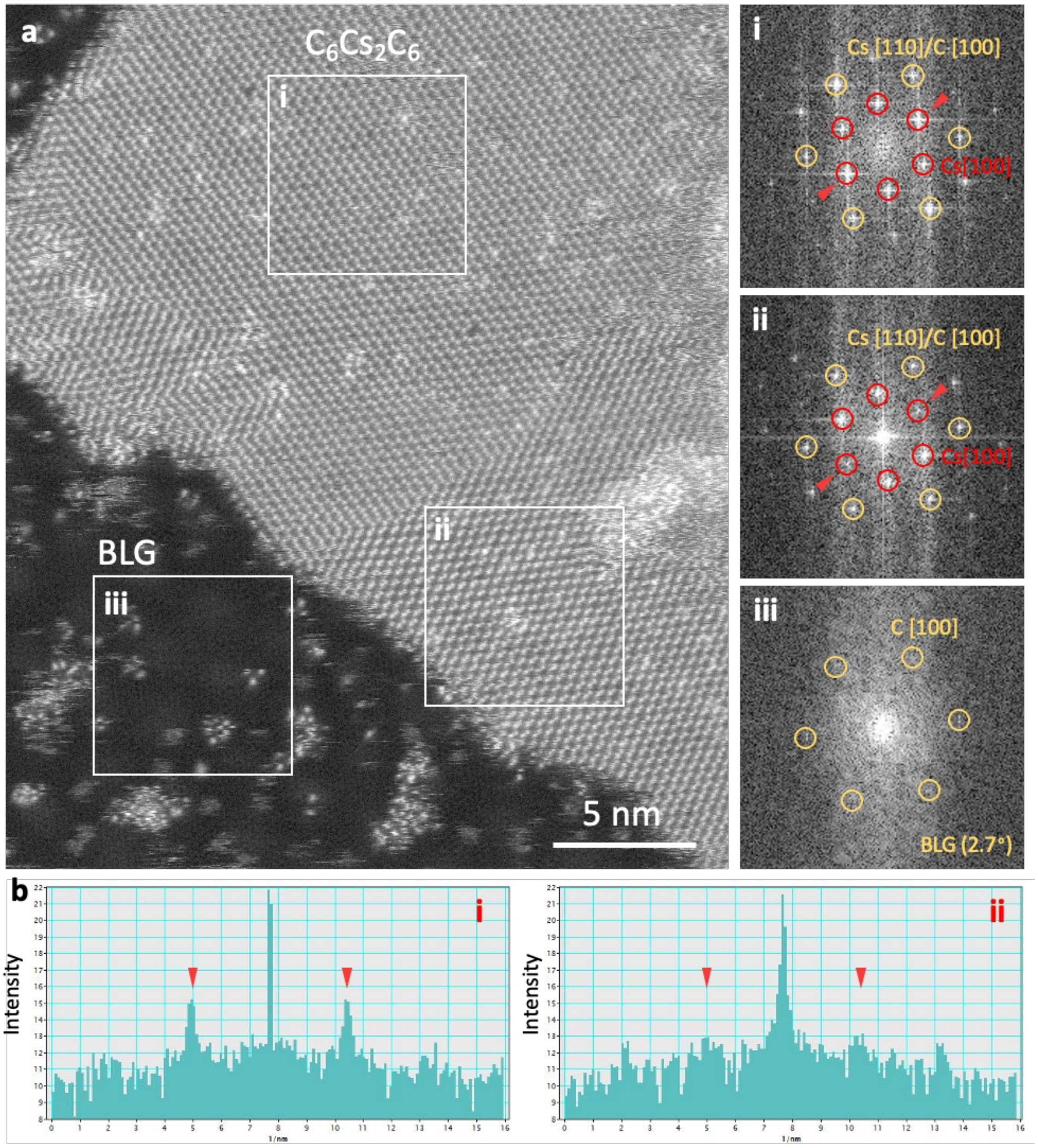

Supplementary Fig. 4 | STEM image of Cs intercalated BLG. a, The original ADF image of Figure 1c shows BLG with a few isolated Cs atoms anchored on the left side, allowing us to determine a twist angle of 2.7° between the two graphene layers from the FFT pattern (iii). On the right side of the ADF image, a large and continuous domain of Cs is observed, displaying a honeycomb lattice with hexagonal symmetry, as confirmed by the FFT pattern (i). The lower right domain of Cs exhibits a laterally displaced configuration, resulting in weaker intensity in one of the [100] spots in the corresponding FFT pattern (ii), as indicated by the red arrows. **b,** The intensity profile of the FFT patterns (i) and (ii) is shown along the direction indicated by the red arrows.

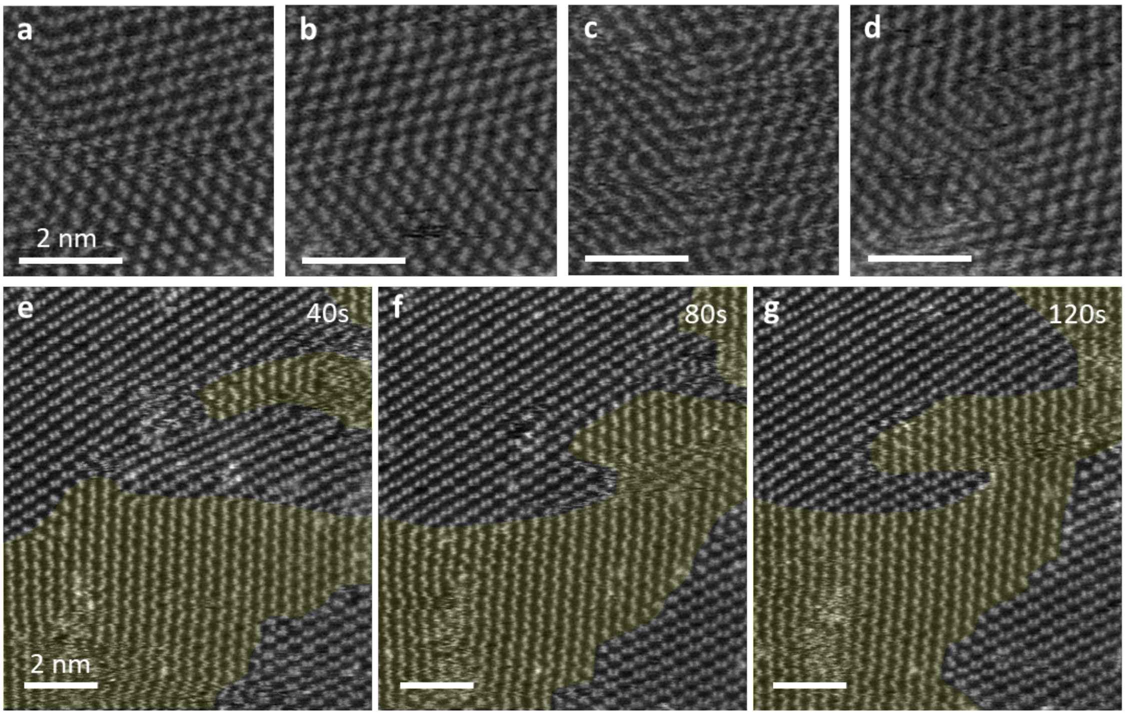

Supplementary Fig. 5 | Characterization of Cs bilayer intercalation in BLG. a-d, ADF images of Cs intercalation in BLG showing Cs domains with varying degree of lateral distortion along different directions. **e-g,** Consecutive ADF images of Cs intercalation in BLG. The yellow-colored Cs domain exhibits a honeycomb structure different from the structure of the surrounding domain. The observed changes in stacking within colored domain are attributed to the lateral displacement of the intercalated Cs atoms.

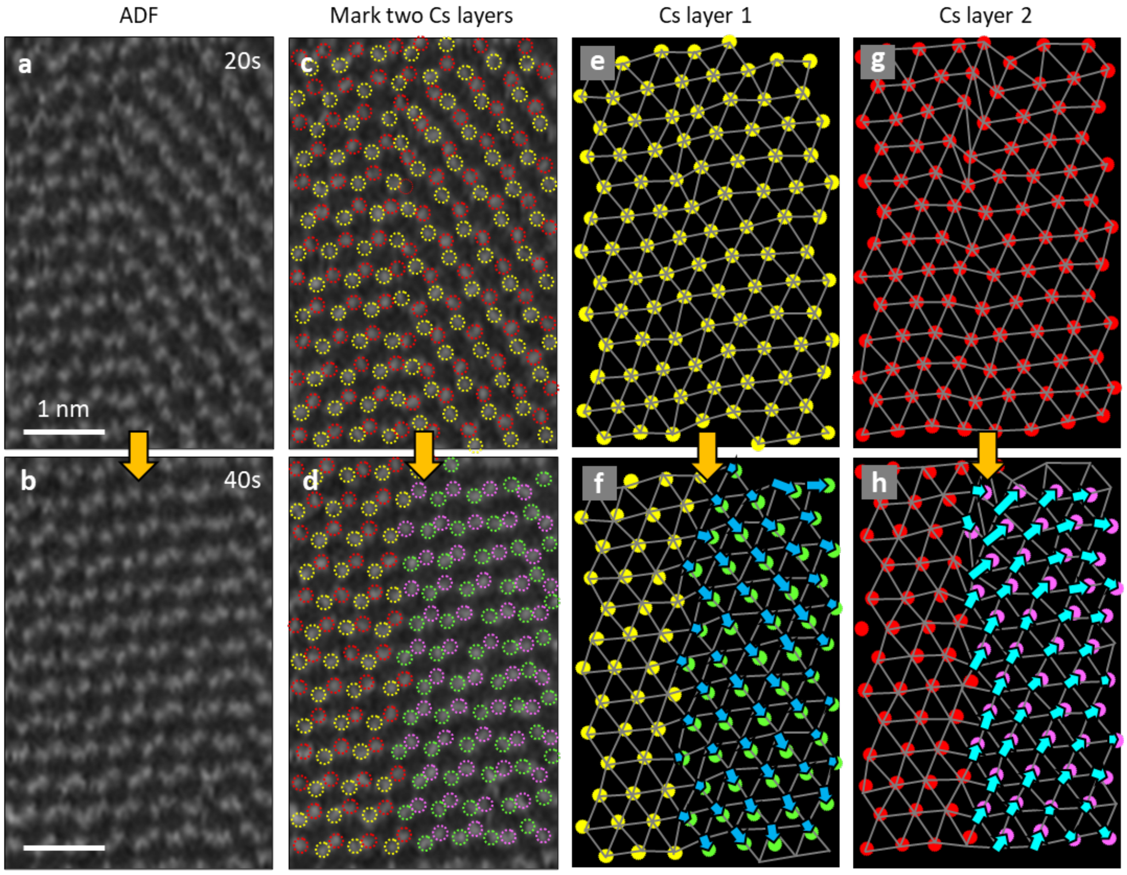

Supplementary Fig. 6 | Restructuring the Cs bilayer domains. a, b, Sequential ADF images of Cs bilayer. **c,** The ADF shown in panel **(a)** in which the Cs atoms are marked by yellow and red dotted circles for Cs layers 1 and 2, respectively. **d,** The ADF image shown in panel **(b)** in which displaced Cs atoms in layers 1 and 2 are marked by green and purple dotted circles, respectively. **e, f,** The corresponding atomic positions of Cs atoms in layer 1 from panels **(c)** and **(d)**, respectively. The gray frame overlay in panel **(f)** shows the atomic positions of Cs in **(e)**. The blue arrows indicate the atomic displacement vectors of Cs in layer 1. **g, h,** The corresponding atomic position of Cs atoms in layer 2 from panels **(c)** and **(d)**, respectively. The cyan arrows indicate the atomic displacement vectors of Cs in layer 2.

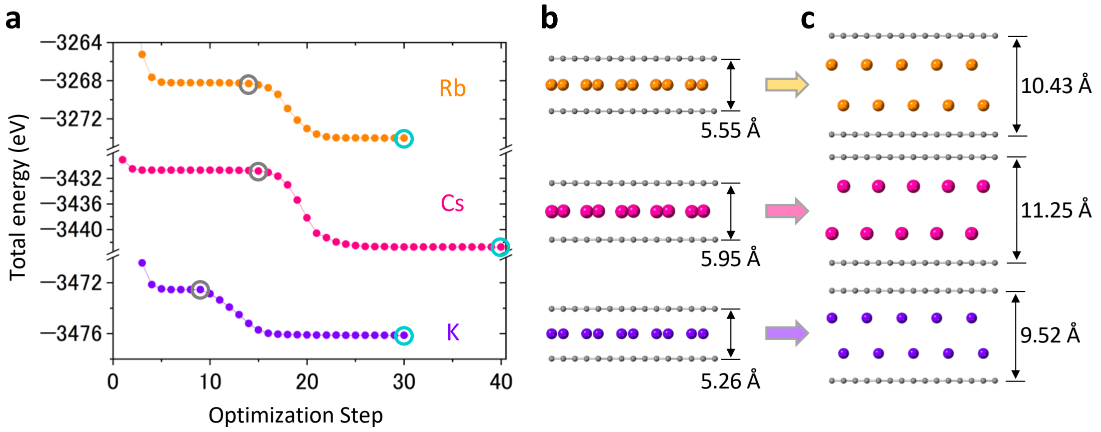

Supplementary Fig. 7 | Geometry optimization for the $C_6K_2C_6$, $C_6Rb_2C_6$, and $C_6Cs_2C_6$. a, The calculated total energy corresponding to the geometry optimization steps for K, Rb, and Cs intercalated in bilayer graphene (BLG). **b,c,** The atomic models corresponding to the first and second optimized plateau, marked by grey and cyan circles, respectively. The total energy is lower when the alkali metal layers are separated at different heights.

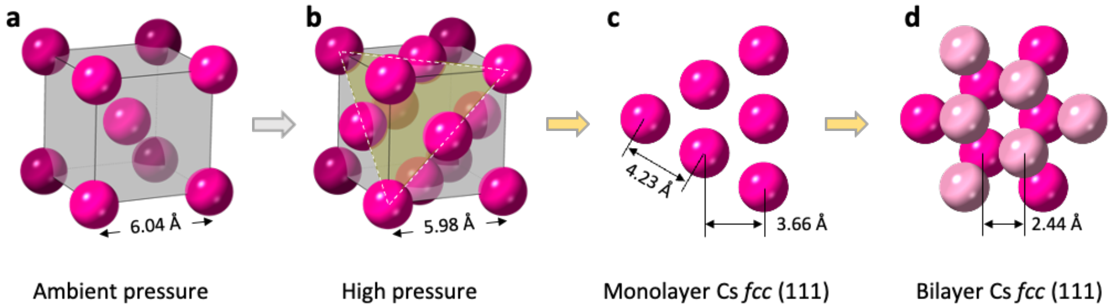

Supplementary Fig. 8 | Atomic structure of Cs crystal at ambient pressure and high-pressure conditions. a, Atomic model of Cs crystal at ambient pressure, displaying a *bcc* structure with a lattice constant of 6.04 Å. **b,** Atomic model of Cs crystal at high pressure (42.2 kbar), illustrating a *fcc* structure with a lattice constant of 5.98 Å. **c,** Atomic model of the high-pressure *fcc* phase Cs crystal viewed in the (111) direction. **d,** Atomic model of the (111) *fcc* phase Cs bilayer, which is identical to the (001) *hcp* Cs bilayer. The atomic layer of *fcc*(111) surface exhibits a 3-fold (actually 6-fold, hexagonal) symmetry, corresponding to one of the *hcp* layers. Consequently, the bilayer of *hcp* structure displays a hexagonal pattern. The *fcc* Cs crystal has a lattice constant of 5.98 Å, while the Cs-Cs

distance in the (111) plane is 4.23 Å. Therefore, the shortest Cs-Cs distance in the *hcp* Cs bilayer, when projected, is calculated to be 2.44 Å, which closely matches the Cs bilayer distance observed in this our STEM images. This finding confirms that spatial confinement between BLG layers provides a two-dimensional (2D) space for Cs atoms to stack and form a high-pressure phase with a bilayer configuration.

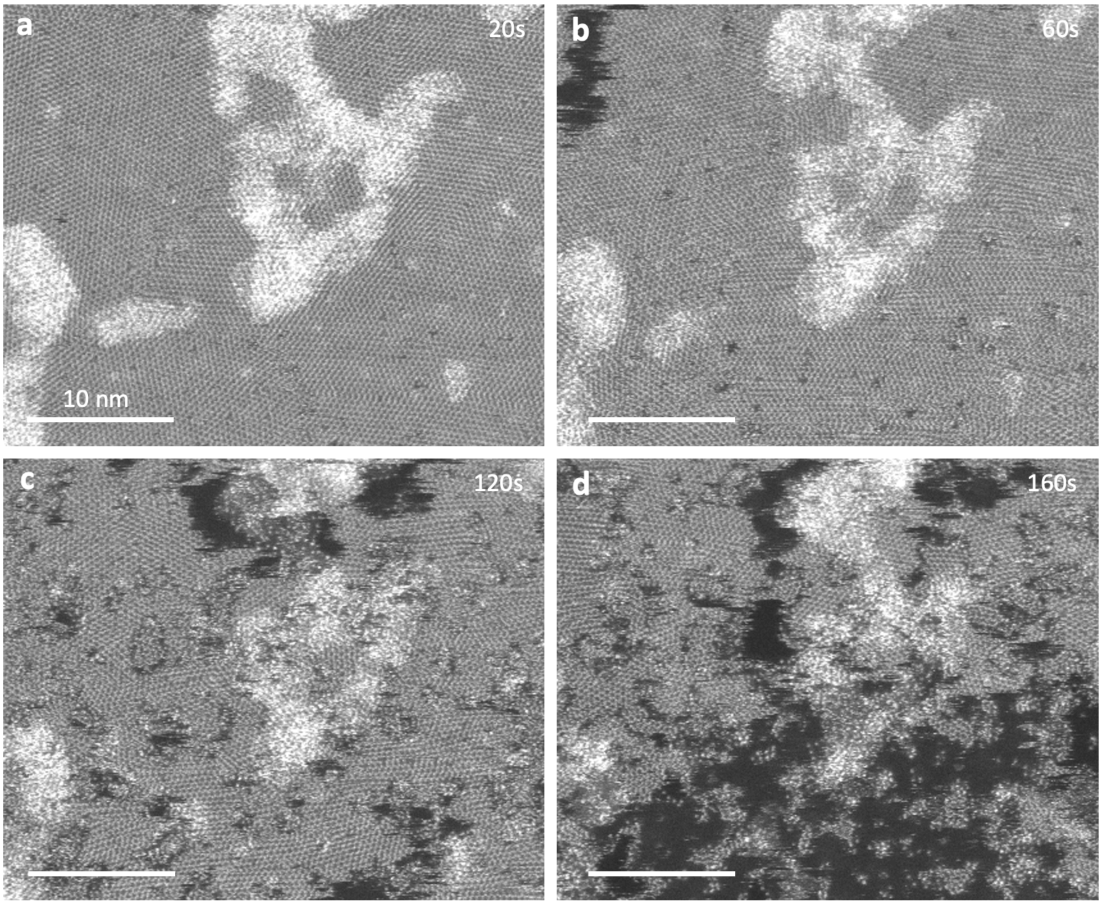

Supplementary Fig. 9 | E-beam irradiation induced damages to the intercalated Cs bilayer. (a-d) Sequential ADF image of Cs sustained under the scanning e-beam observation for more than 2 minutes, with damaged Cs confined within the BLG domain, suggesting as an intercalated structure.

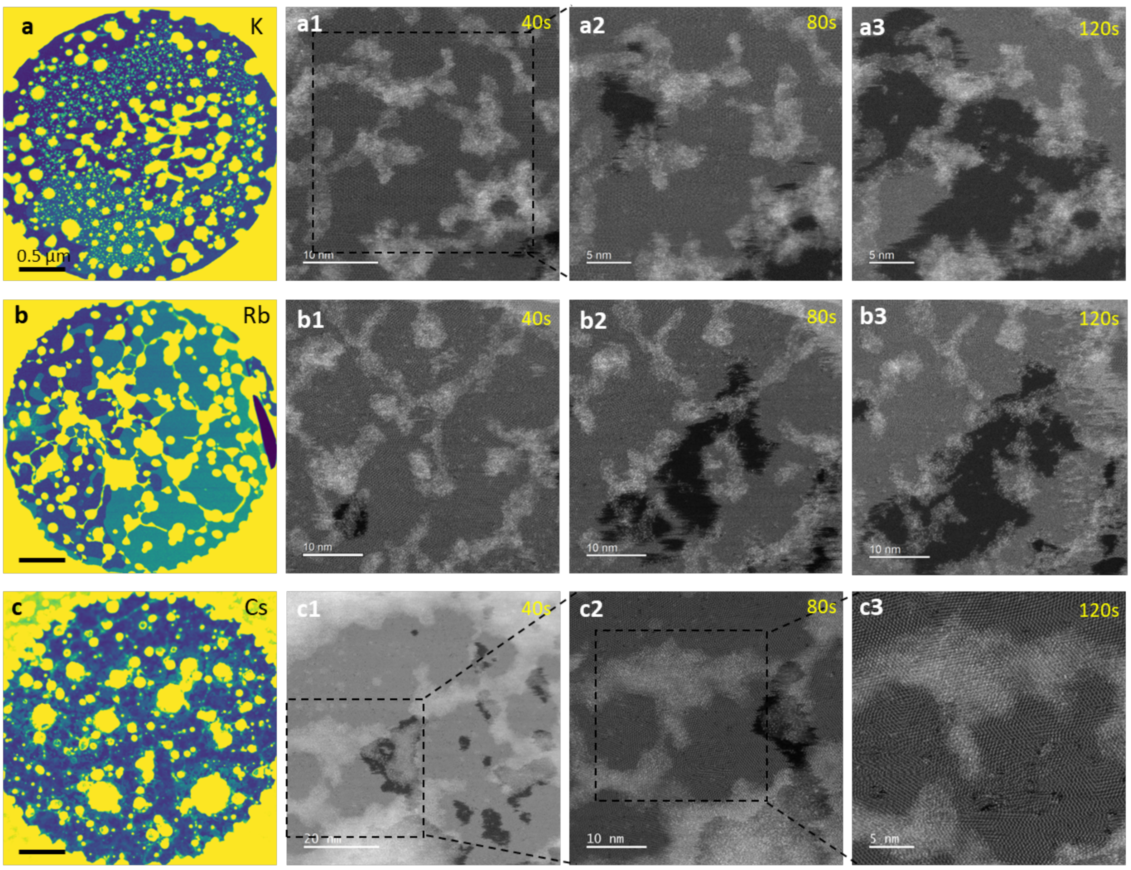

Supplementary Fig. 10 | Characterization of K, Rb, and Cs intercalation in BLG. a, Low-magnification ADF image of K intercalation in BLG. **a1-a3,** Consecutive ADF images of K-intercalated BLG showing rapid damage to the K bilayer during e-beam scanning. **b,** Low-magnification ADF image of Rb intercalation in BLG. **b1-b3,** Consecutive ADF images of Rb-intercalated BLG showing rapid damage to the Rb bilayer during e-beam scanning. **c,** Low-magnification ADF image of Cs intercalation in BLG. **c1-c3,** Consecutive ADF images of Cs-intercalated BLG showing the relatively stable Cs bilayer compared to K and Rb during e-beam scanning. Images **(a), (b),** and **(c)** are colorized using the look-up table (LUT) in ImageJ software.

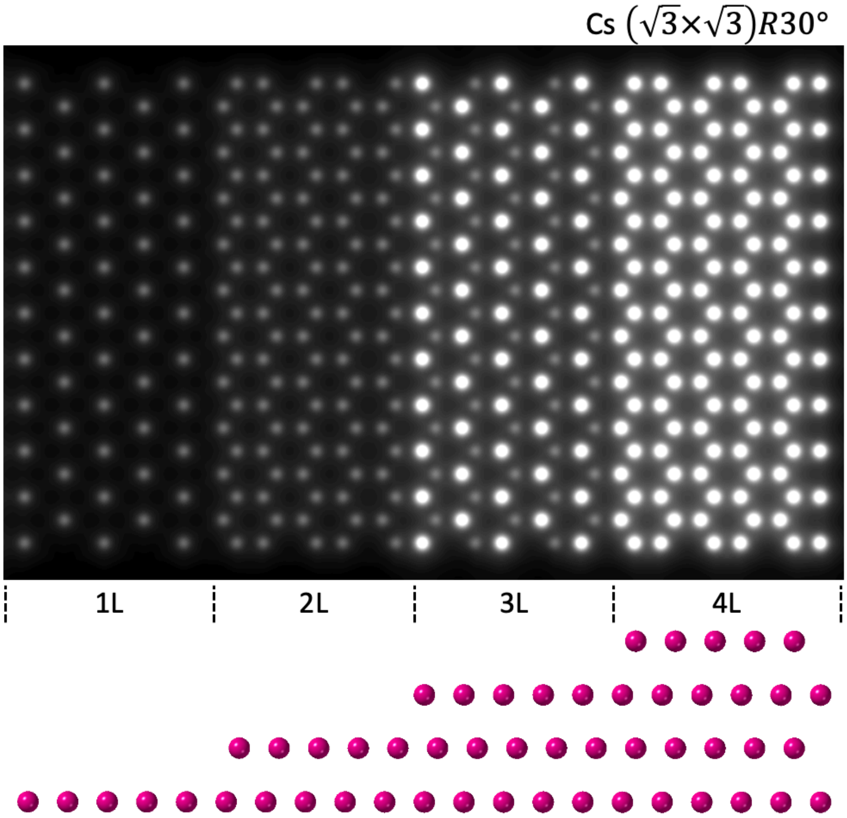

Supplementary Fig. 11 | Structural comparison of intercalated Cs in multilayer form. (a) STEM simulation and cross-section atomic model for 1-4 layers of Cs with a $(\sqrt{3}\ \times\sqrt{3}\ )R30^{\circ}$ structure. (b) STEM simulation for the stacking of two Cs bilayers with a twist angle of 10°.

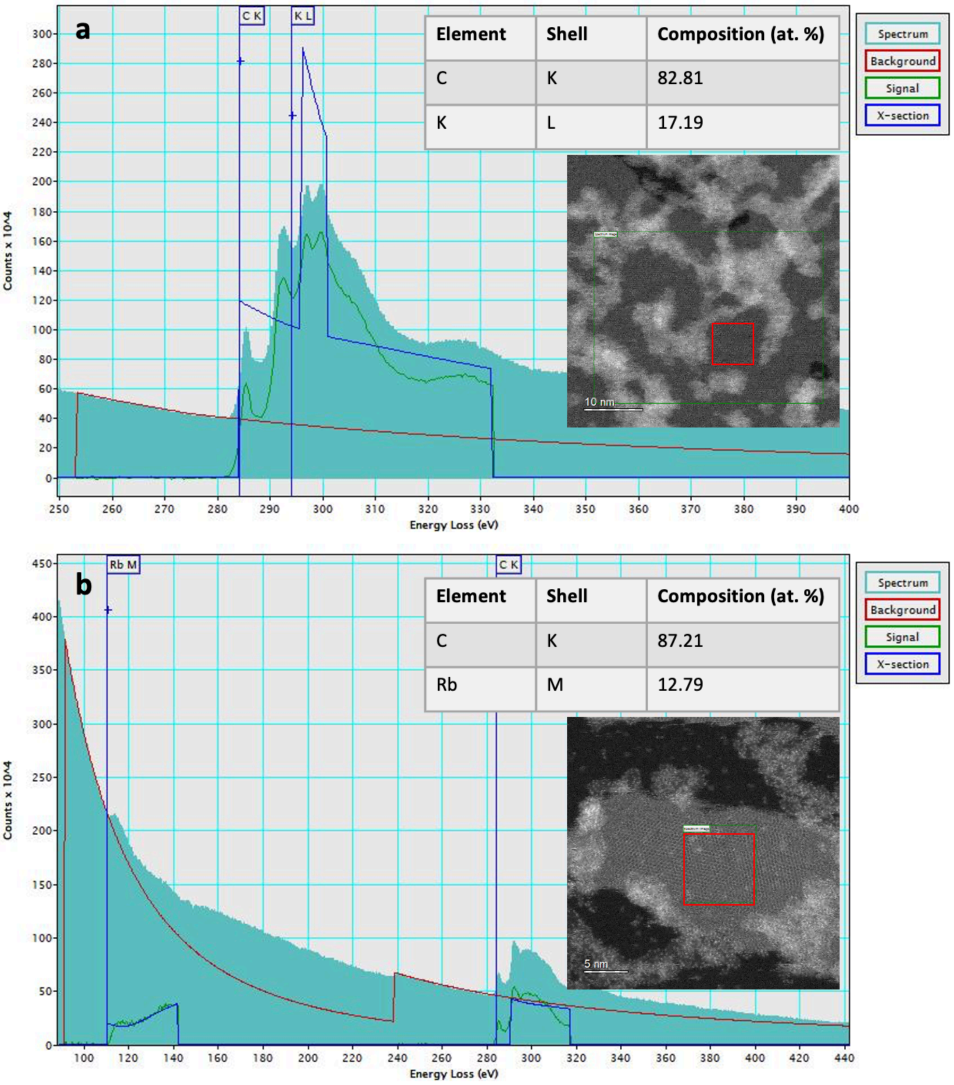

Supplementary Fig. 12 | EELS quantification for the $C_6K_2C_6$ and $C_6Rb_2C_6$. (a) In $C_6K_2C_6$, the calculated atomic ratio for C is 82.81 at%, and for K, it is 17.19 at%. The K:C ratio is slightly higher than the expected value (1:6) due to the overlapping of the K L-edge and the C K-edge, resulting in a higher background signal for K. (b) In $C_6Rb_2C_6$, the calculated atomic ratio for C is 87.21 at%, and for Rb, it is 12.79 at%, which is consistent with the $C_6Rb_2C_6$ structure.

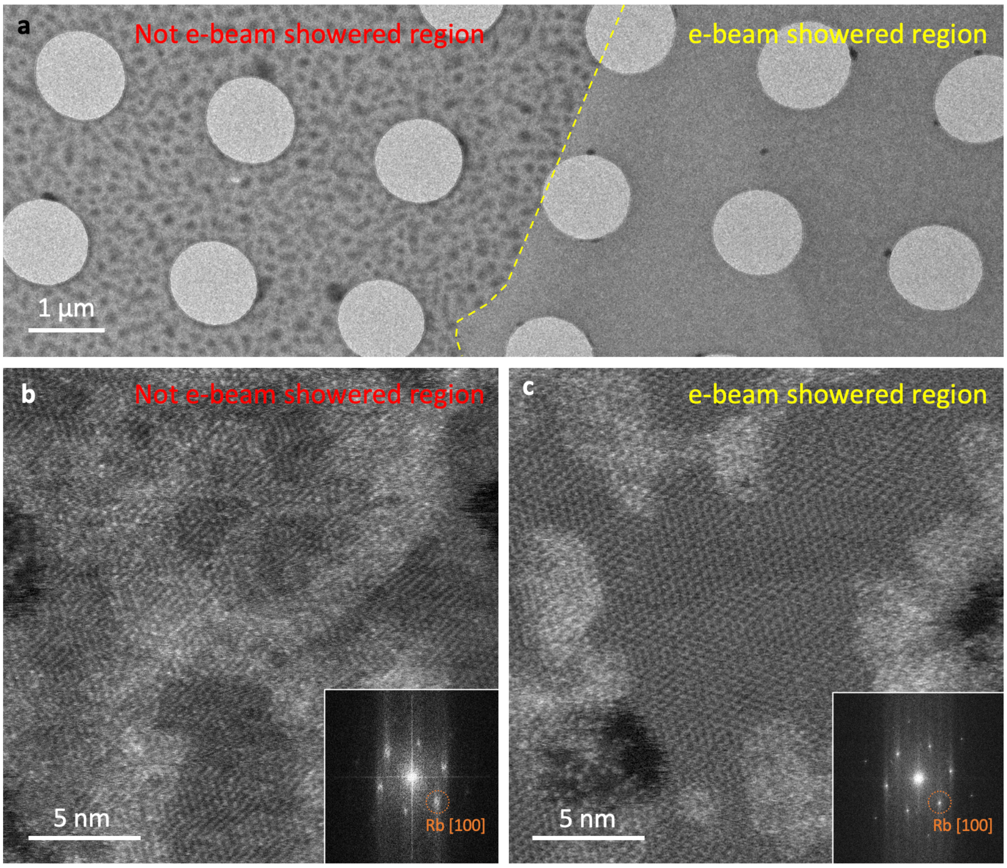

Supplementary Fig. 13 | Removal of surface deposited amorphous AM by e-beam. (a) Low-magnification TEM image of the Rb-intercalated BLG sample. The area to the right side from the yellow dash line depicts the region exposed to the e-beam shower, resulting the removal of most surface-deposited materials. (b) ADF image captured from the non-e-beam showered region, where the crystalline Rb structure is obscured by surface contamination. (c) ADF image obtained from the e-beam showered region, revealing a clear resolution of the crystalline intercalated Rb structure.

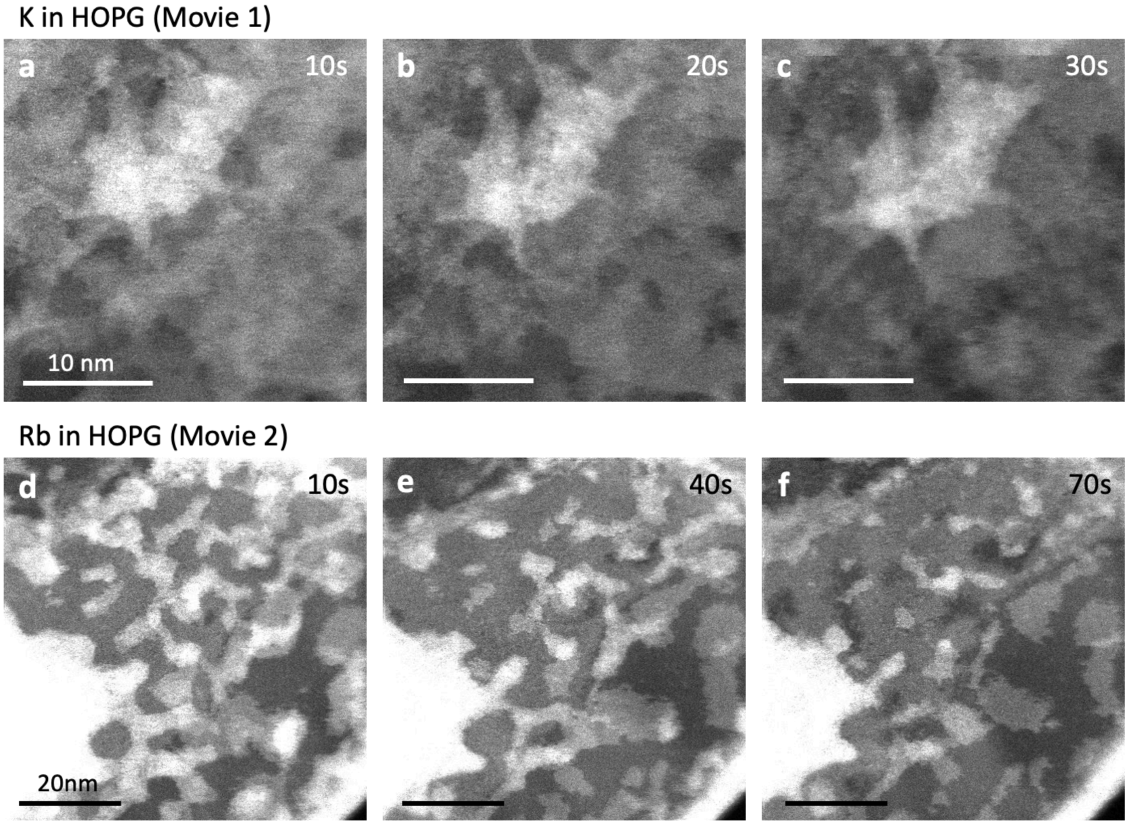

Supplementary Fig. 14 | STEM characterization of the K and Rb intercalated HOPG. (a-c) Sequential ADF images of K-intercalated HOPG extracted from Movie 1. (d-f) Sequential ADF images of Rb-intercalated HOPG extracted from Movie 2. In both samples, we observe the removal of the layered structure due to e-beam scanning, suggesting the presence of mobile layered structures, likely corresponding to single-layer K and Rb intercalated within the graphite layers.

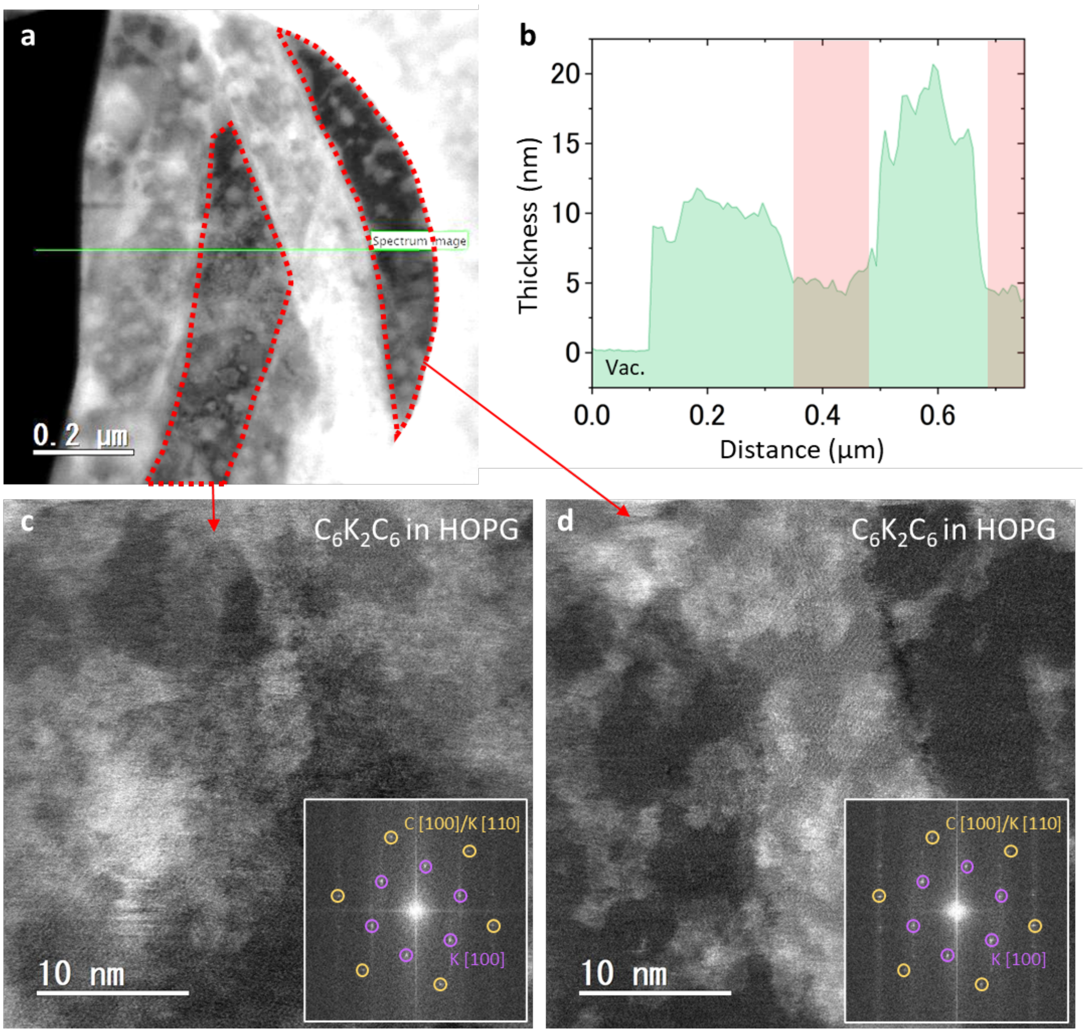

Supplementary Fig. 15 | Characterization of K intercalation in few layer HOPG. a, ADF image of K-intercalated few layer HOPG. The green line indicates the EELS line scan used to measure the specimen's thickness. The red dotted regions indicate areas where $C_6K_2C_6$ structure can be observed. **b,** Thickness measurement of Cs-intercalated HOPG calculated from the zero-loss spectrum along the green line in **(a)**. The thickness was determined using the log-ratio equation $t/\lambda=\ln(I_t/I_0)$, where the t is the specimen thickness and λ is the local inelastic mean free path. It represents the total number of electrons in the EELS spectrum, while I_0 corresponds to the number of electrons with no energy loss (the zero-loss peak). I_t is the sum of I_0 and the intensity of the inelastic peak ($I_{inel.}$). $I_t = I_0+I_{inel}$. The region where the $C_6K_2C_6$ structure can be found has an approximate thickness of 5 nm. **c,** ADF image taken from the left red-dotted region in **(a)**. **d,** ADF image taken from the right red-dotted region in **(a)**.

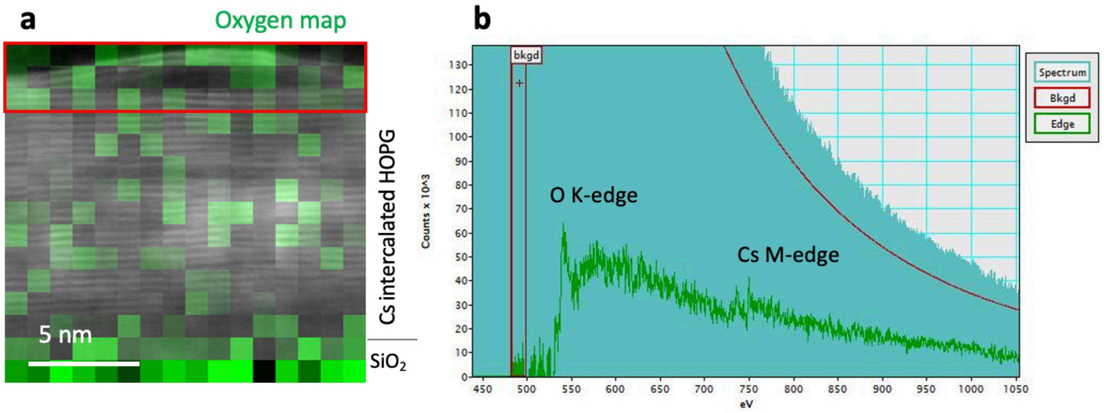

Supplementary Fig. 16 | EELS characterization to the oxidization behavior in the cross-sectional sample. (a) EELS colored mapping for Cs-intercalated HOPG. The green pixels represent oxygen intensity mapping. The sample surface contains a high density of oxygen, and some oxygen is also present in the cross-sectional plane, likely due to the sample preparation process. (b) EELS profile extracted from the red box in (a).
