# Supplementary material for: Alkali metal bilayer intercalation in graphene
Source: Nat Commun. 2024 Jan 24;15:425. doi: 10.1038/s41467-023-44602-3 (PMC11258350; doi:10.1038/s41467-023-44602-3)
Supplement: Supplementary file 3 — Description of Additional Supplementary Files [file 41467_2023_44602_MOESM3_ESM.pdf]

Description of Additional Supplementary Files

File Name: Supplementary Movie 1
Description: Sequential STEM image of K intercalation in HOPG.

File Name: Supplementary Movie 2
Description: Sequential STEM image of Rb intercalation in HOPG.
